# mmCSM-AB: guiding rational antibody engineering through multiple point mutations

**DOI:** 10.1093/nar/gkaa389

**Published:** 2020-05-20

**Authors:** Yoochan Myung, Douglas E V Pires, David B Ascher

**Affiliations:** Computational Biology and Clinical Informatics, Baker Institute, Melbourne, VIC 3004, Australia; Structural Biology and Bioinformatics, Department of Biochemistry and Molecular Biology, Bio21 Institute, University of Melbourne, Parkville, VIC 3052, Australia; Computational Biology and Clinical Informatics, Baker Institute, Melbourne, VIC 3004, Australia; Structural Biology and Bioinformatics, Department of Biochemistry and Molecular Biology, Bio21 Institute, University of Melbourne, Parkville, VIC 3052, Australia; School of Computing and Information Systems, University of Melbourne, Parkville, VIC 3052, Australia; Computational Biology and Clinical Informatics, Baker Institute, Melbourne, VIC 3004, Australia; Structural Biology and Bioinformatics, Department of Biochemistry and Molecular Biology, Bio21 Institute, University of Melbourne, Parkville, VIC 3052, Australia; Department of Biochemistry, University of Cambridge, Cambridge CB2 1GA, UK

## Abstract

While antibodies are becoming an increasingly important therapeutic class, especially in personalized medicine, their development and optimization has been largely through experimental exploration. While there have been many efforts to develop computational tools to guide rational antibody engineering, most approaches are of limited accuracy when applied to antibody design, and have largely been limited to analysing a single point mutation at a time. To overcome this gap, we have curated a dataset of 242 experimentally determined changes in binding affinity upon multiple point mutations in antibody-target complexes (89 increasing and 153 decreasing binding affinity). Here, we have shown that by using our graph-based signatures and atomic interaction information, we can accurately analyse the consequence of multi-point mutations on antigen binding affinity. Our approach outperformed other available tools across cross-validation and two independent blind tests, achieving Pearson's correlations of up to 0.95. We have implemented our new approach, mmCSM-AB, as a web-server that can help guide the process of affinity maturation in antibody design. mmCSM-AB is freely available at http://biosig.unimelb.edu.au/mmcsm_ab/.

## INTRODUCTION

The ability of antibodies to selectively and specifically bind tightly to targets and sites considered undruggable, has seen them become a major focus of therapeutic and diagnostic applications in a wide range of diseases. This specificity can be so highly tuned that they can be used to even selectively recognize a unique missense mutation, leading to their successful application in personalized medicine (1,2). As antibody therapies become more common, new approaches to more quickly and cheaply optimize the binding affinity and specificity, known as antibody maturation, are increasingly necessary. While experimental approaches to explore antibody binding space have become more efficient, previously successful efforts have shown that at least two single-point mutations are generally needed, which do not necessarily lie only in the complementarity-determining regions (CDRs) (3). Exploring all possible permutations and combinations of mutations has therefore remained a bottleneck in the antibody development pipeline.

Increasing computational power has led to a number of different approaches to guide the rational engineering of antibody binding and specificity. Initial approaches used a range of techniques, including homology modelling (4), protein–protein docking (5–7), energy functions (8–10) and more recently machine learning-based approaches (11–13). While these have been successfully used in the development of a number of clinical antibodies, they have generally been limited to the analysis of single-point missense mutations, and have been shown to be only weakly correlated with experimentally measured changes.

We have previously shown that by using graph-based signatures to represent the wild-type residue environment we can accurately predict the effects of mutations on protein folding, stability (14–16), dynamics (17), function (18) and interactions (15,19–25). These have provided insights into genetic diseases (26–32), drug resistance (33–42), pharmacokinetics (43–46) and rational protein engineering (47). Extending this to look at antibody engineering, we developed mCSM-AB2 (25), which was able to more accurately predict the effects of single-point missense mutations on antibody binding affinity. However, at the time the representations used by mCSM-AB2 and the amount of data available, still limited its ability to screen for the additive or synergistic effects of combinations of mutations.

Here, we present a new approach, mmCSM-AB, as a web-server that enables rapid and deep evaluations of combinations of multiple mutations in antibody-antigen complexes using graph-based signatures, sequence- and structure-based information. mmCSM-AB models were trained using single-point mutations and the effects of multiple mutations were assessed, outperforming other available tools across our validation set of experimentally measured changes with double to 14 mutations. mmCSM-AB will help to guide rational antibody engineering by analysing the effects of introducing multiple mutations on binding affinity.

## MATERIALS AND METHODS

### Datasets

**Figure 1.**
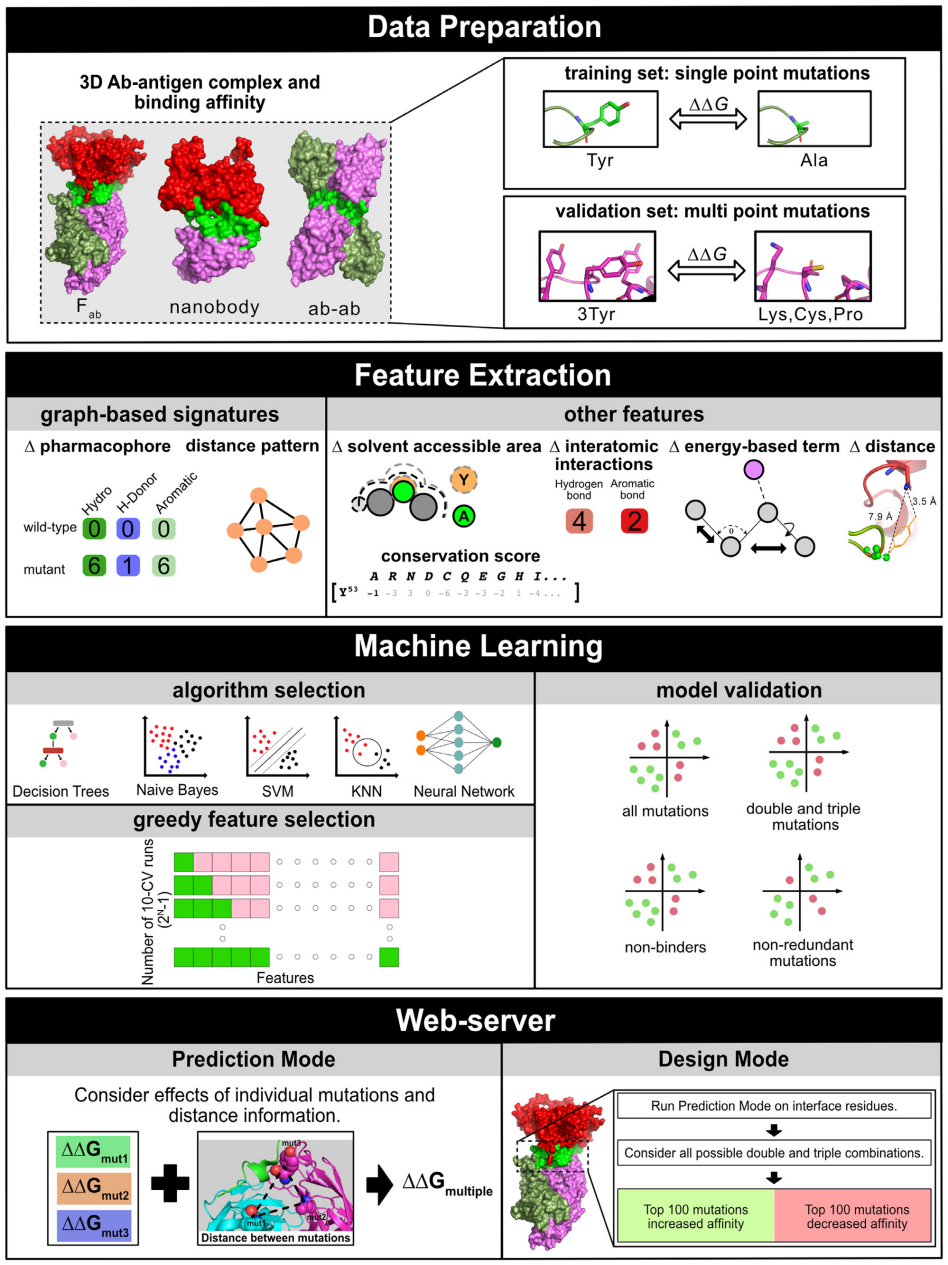
mmCSM-AB workflow. Method development can be divided into four steps. During data preparation, 905 single point mutation and 242 multi-point mutations across 62 complexes (50 Fab, 3 nanobody, 1 monobody,1 Ab-Ab and 7 others described in Supplementary Table S1) were collected. During feature extraction, the curated 3D structures were used to calculate graph-based signatures as well as complementary structure- and sequence-based attributes. On the next step, these were provided as evidence to train and test supervised learning algorithms. Greedy feature selection was performed to optimize performance on multiple mutations and reduce dimensionality. On the last step, the best performing model was implemented into three prediction modes; the ‘Prediction mode’ for running user-specified mutations and the Antibody and Antigen Design modes, for systematically assessing permutation of multiple mutations on the antibody and antigen, respectively, at the binding interface.

Effects of single-point mutations on antibody-antigen binding affinity (in terms of *K*_D_, given in molar) were collected for 62 complexes (Figure 1 and Supplementary Table S1) with known structures available at the Protein Data Bank. The quality of 3D structures was reviewed with the resolution and R-value (Figure S1) from the wwPDB X-ray structure validation report. Mutation information for wild-type and mutant was compiled from three different resources: AB-BIND (48), PROXiMATE (49) and SKEMPI2.0 (50).

Since the binding affinity of non-binders cannot be accurately measured, non-binders were excluded from our training dataset. This generated a final dataset of 905 single missense mutations, and 242 multiple missense mutations with experimentally measured changes in binding affinity. Binding affinities of wild-type and mutant structures were converted to binding energy (ΔG, given in Kcal/mol) (Equation 1) and the effects of mutations expressed in terms of the change in binding energy (ΔΔG, Equation 2).

(1) $$\begin{equation*}\Delta G\; = \;RT\ln\left( {{K_{\rm{D}}}} \right)\end{equation*}$$

(2) $$\begin{equation*}\Delta \Delta G\; = \Delta {G_{{\rm wild}}}\; - \;\Delta {G_{{\rm mutant}}}\end{equation*}$$

#### Training set

The 905 single point mutation training set presented a skewed distribution, with an average ΔΔ*G* of –1 kcal/mol (Supplementary Figure S2A). To avoid potential bias in our machine-learning models, we also included the hypothetical reverse mutations, as previously proposed (17,20,23,51). Only reverse mutations with a measured effect in affinity below 2 kcal/mol were considered, to avoid situations where the reverse mutation could potentially compromise binding, with a total of 735 reverse mutations being modelled. This resulted in a final training data-set of 1640 mutations with associated changes in binding affinity.

#### Blind-test set

To evaluate our approach on multiple point mutations, the curated set of 242 experimentally characterized multiple mutants was used (Supplementary Figure S2B). This included multi-point mutations ranging from 2 to 14 mutations (Supplementary Figure S2C). Based on the proportion of the number of multiple mutations, the 242 blind-test set was further divided into two subsets; 101 double and triple mutations and 242 all multiple missense mutations and assessed separately.

#### Assessing additive and synergistic multiple point mutations

To explore the role of additive and synergistic effects across our dataset, we identified a set of 38 multiple point mutations, where each individual mutation had been experimentally characterized as a single-point missense mutation. Additive mutations were defined as when the sum of the individual mutations was within 1 kcal/mol of the multiple-point mutation. We identified 24 additive and 14 synergistic mutations.

#### Non-binder dataset

During data curation we identified 47 sets of multiple mutations, which when experimentally evaluated completely disrupted antigen binding. These were excluded from the training and test sets, but used for further evaluation of the mmCSM-AB models.

#### Validation set

We collected an additional 59 experimentally characterized multiple mutation complexes from a previous study benchmarking the performance of available methods in predicting effects of mutations on antibody-antigen affinity (52). This dataset was not present in our bind-test set, and was therefore used as a validation set to compare the performance of mmCSM-Ab with other methods.

All datasets used are available for download at http://biosig.unimelb.edu.au/mmcsm_ab/datasets/.

### Mutation modelling and feature engineering

#### Modelling mutant structures

Modeller (v.9.21) (53) was used to build single point mutants as well as multiple mutants incrementally. Structures were submitted for energy minimization using FoldX (54).

#### Graph-based signatures

The mCSM graph-based signatures have been widely adopted to capture both the geometry and physicochemical properties of wild-type residue environment using the cutoff scanning matrix algorithm and the resulting pharmacophore changes upon mutation (15). mCSM signatures were the main feature class used to train the mmCSM-AB predictive method.

#### Energetic terms

FoldX was used to calculate interaction energies for both wild-type and modelled mutant structures.

#### Interface properties

To capture changes in interaction networks upon mutation, all non-covalent interactions in the wildtype and mutant structures were calculated using Arpeggio (55). The difference in the solvent accessible surface area between wild-type and mutant structures was calculated using DSSP (56). Additionally, the distance from the mutated sites to binding partner was also calculated.

#### Evolutionary scores

In order to capture residue conservation, we employed different evolutionary scoring measures. These include Position Specific Scoring Matrices (PSSMs) calculated from multiple sequence alignments using PSI-BLAST (57) on the non-redundant UniProtKB/SwissProt database and substitution sores from PAM30.

#### Mutation distances

To account for potential synergistic and compensatory effects of mutations, we also included information on the distances between the individual mutations.

### Machine Learning

A range of supervised learning algorithms for regression currently available within the scikit-learn Python library were evaluated. These included Random Forest, Extra Trees, Gradient Boost, XGBoost, SVM and Gaussian Process. The best performing model was selected based on Pearson's correlation coefficient and Root Mean Squared Error (RMSE), evaluated under different cross-validation schemes (with 10 bootstrap repetitions), as well as blind tests. The best performing algorithm was ExtraTrees. In order to reduce dimensionality and improve performance, feature selection was carried out in an incremental stepwise greedy approach.

## WEB SERVER

mmCSM-AB was developed using Materialize 1.0.0 and Flask 1.0.2, and hosted on an Apache2 Linux server. This webserver is freely available at http://biosig.unimelb.edu.au/mmcsm_ab.

### Input

mmCSM-AB can analyse the effects of introducing multiple point mutations on antibody-antigen binding affinity. It can be used to either predict the effects of a known mutation via Prediction Mode, or systematic exploration of all potential multiple mutations at the interface to guide rational antibody engineering via Design mode (Supplementary Figure S3).

The server requires the user to provide (i) an antibody-antigen PDB structure either in a PDB file or PDB accession code; (ii) for Prediction Mode, provide a multiple mutation denoted by list of point mutations separated by semicolons, with mutations specified as the chain ID, wild-type residue one-letter code, residue number, and mutant residue one-letter code. Alternatively, users can upload a list of multiple mutations as a text file. Design mode automatically considers all possible combinations of double- and triple-point mutations of residues on either the antibody or the antigen side of the interface.

### Output

The Prediction Mode result page consists of three sections. The mutation table shows predicted ΔΔ*G* (in kcal/mol) of a given multiple mutation and complementary details such as distance among single point mutations and distance to interface (Supplementary Figure S4). If the user provides more than one multiple mutation, the order of each result in the mutation table will be the same order as the given mutation list. Users can check wild-type atomic interactions of each of single point mutations via 3D molecular viewer, implemented using an NGL plugin (58). All results in the data table and 3D visualization can be downloaded as comma-separated file (CSV) and PyMOL session file (PSE), respectively.

The output of Design Mode shows the top 100 increasing/decreasing multi-point mutations at the binding interface of a given antibody-antigen complex (Supplementary Figure S5). Users need to select between stabilizing and destabilizing mutations in the mutation table to browse corresponding mutations. Similar to the Prediction Mode, each row of the mutation table provides information about antibody annotation, distance to interface and predicted ΔΔG. By selecting the radio box in the first column, users can visualize the wild-type structure of each multi-point mutation in the 3D visualization section. All results of the mutation table are downloadable as a CSV file.

## VALIDATION

### Performance on cross-validation

In order to build a robust and reliable model for predicting the effects of mutations on antibody binding affinity, mmCSM-AB was trained using different stratified cross-validation approaches. We achieved Pearson's correlations up to 0.70 (RMSE = 1.03 kcal/mol) on 5-, 10- and 20-fold cross validation. We further validated the model, by performing a low redundancy leave-one-complex out validation, achieving a Pearson's correlation up to 0.64 (RMSE = 1.10 kcal/mol). Following feature selection using a greedy algorithm, we were left with a total of 83 features. Interestingly, the only features selected for the final model were the graph-based structural signatures, the evolutionary score and the Arpeggio calculated interactions.

### Performance on multi-point mutations

**Figure 2.**
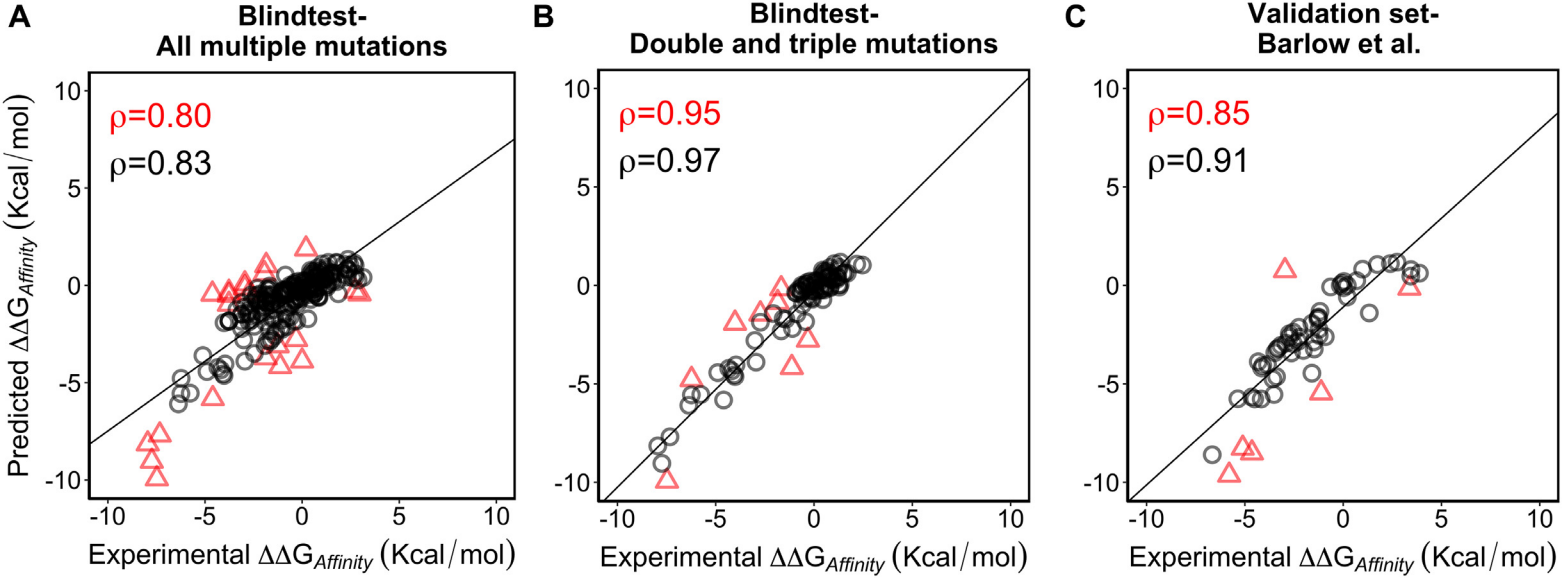
Performance of mmCSM-AB on multiple mutations. The figure depicts the performance of the final mmCSM-AB model on independent blind tests. The method achieved a Pearson's correlation of 0.80 (RMSE = 1.30) on all multiple missense mutations (242 in total) (**A**) and 0.95 (RMSE = 0.91) on double and triple mutation (101 in total) (**B**). Performance increases to 0.95 and 0.97, respectively, after 10% of outliers are removed. On an additional validation set comprised of 59 multiple mutations, mmCSM-AB achieved a correlation of 0.85 (RMSE = 1.66) (**C**), increasing to 0.91 across 90% of the data.

Our final mmCSM-AB model achieved a Pearson's correlation of 0.95 (RMSE = 0.91 kcal/mol, Figure 2) and 0.80 (RMSE = 1.30 kcal/mol, Figure 2) on the 101 double and triple mutations and the overall 242 multiple mutations, respectively (Table 1). Comparing the performance of mmCSM-AB to 18 alternative approaches, mmCSM significantly outperformed all methods across both mutation sets (*P*-value <0.01, Fisher's *r*-to-*z* transformation; Table 1). The inclusion of the hypothetical reverse mutations led to a significant improvement in performance. In particular, inclusion of hypothetical reverse mutations resulted in a final model with significantly improved ability to classify mutations as leading to increased binding affinity (Supplementary Table S2).

**Table 1.** Performance comparison of available methods

	Blind-test											
	242 all mutations			101 double and triple mutations			104 unique all mutations			53 unique double and triple mutations		
Method	Pearson (ρ)	Spearman (ρ)	RMSE	Pearson (ρ)	Spearman (ρ)	RMSE	Pearson (ρ)	Spearman (ρ)	RMSE	Pearson (ρ)	Spearman (ρ)	RMSE
mmCSM_AB	0.80	0.78	1.30	0.95	0.89	0.91	0.77	0.72	1.15	0.92	0.84	0.66
mCSM_PPI	0.12***	0.12***	4.13	0.64***	0.36***	2.50	0.29***	0.23***	4.58	0.11***	0.01***	2.39
mCSM_PPI2	0.56***	0.45***	1.88	0.89**	0.76**	1.14	0.25***	0.23***	1.91	0.65***	0.58**	1.18
mCSM_AB	0.47***	0.39***	2.61	0.55***	0.32***	2.99	0.08***	0.01***	3.21	0.07***	0.31***	3.53
mCSM_AB2	0.66***	0.39***	2.84	0.88**	0.61***	3.40	0.45***	0.32***	2.29	0.62***	0.27***	1.91
DiscoveryStudio	0.63***	0.58***	1.83	0.76***	0.56***	1.79	0.48***	0.25***	1.50	0.67***	0.43***	1.27
DFIRE	0.52***	0.53***	1.93	0.70***	0.55***	2.14	0.36***	0.34***	1.52	0.48***	0.34***	1.36
bASA	0.47***	0.54***	71.08	0.68***	0.67***	57.76	0.32***	0.24***	43.20	0.57***	0.53**	50.22
dFoldX	0.45***	0.54***	2.06	0.54***	0.63***	2.20	0.44***	0.31***	1.73	0.68***	0.57**	1.29
SIPPER	0.41***	0.52***	3.59	0.43***	0.53***	3.45	0.36***	0.39***	2.49	0.44***	0.56**	2.26
ZRANK	0.37***	0.42***	7.23	0.40***	0.38***	7.95	0.34***	0.18***	8.78	0.46***	0.21***	10.11
dFIRE	0.33***	0.34***	2.15	0.70***	0.55***	2.14	0.36***	0.34***	1.52	0.48***	0.34***	1.36
PRODIGY	0.32***	0.37***	2.20	0.39***	0.46***	2.60	0.17***	0.16***	1.62	0.47***	0.37***	1.42
FIREDOCK	0.28***	0.28***	5.11	0.30***	0.24***	4.49	0.19***	0.05***	5.61	0.24***	0.09***	4.82
FIREDOCK_AB	0.26***	0.27***	5.52	0.26***	0.21***	4.70	0.19***	0.07***	5.90	0.19***	0.02***	5.17
ROSETTADOCK	0.19***	0.38***	2.32	0.19***	0.24***	2.69	0.22***	0.26***	1.78	0.34***	0.33***	1.47
ZRANK2	0.19***	0.62***	118.44	0.17***	0.67***	178.89	0.26***	0.37***	175.97	0.33***	0.55**	245.93
INSIDE	0.07***	0.01***	2.48	0.11***	0.02***	2.68	0.11***	0.03***	1.68	0.04***	0.15***	1.66
LISA	0.00***	0.04***	2.39	0.15***	0.16***	2.80	0.21***	0.26***	1.93	0.39***	0.49***	2.01

**P-value < 0.01, ***P-value < 0.001; statistical significance of Pearson's correlation coefficient was evaluated by Fisher's *r*-to-*z* transformation (two-tailed) and Spearman's rank-correlation coefficient was converted as described by Walker *et al.* (2003) into Pearson's correlation before we applied the transformation.

For comprehensive reviews of the performance in classifying favourable and unfavourable mutations across available methods, the predicted values from the comparative study (Table 1) were further classified as either increasing (ΔΔG > 0.5 Kcal/mol) or decreasing (ΔΔ*G* ≤ –0.5 kcal/mol) binding affinity. Using mmCSM-AB to classify mutations into these two categories, we achieved a Mathew's Correlation Coefficient (MCC) of 0.67 and *F*1-score of 0.89. Supplementary Figure S6 shows the corresponding ROC curves, with our predictor outperforming other methods achieving an Area Under the ROC Curve (AUC) of 0.94.

To evaluate whether the performance relies on the training dataset, we filtered out 53 out of 101 double and triple mutations and 104 out of the overall 242 multiple mutations where none of the mutations was present in the training dataset. mmCSM-AB achieved a Pearson's correlation of 0.92 (RMSE = 0.66 kcal/mol, Table 1) and 0.77 (RMSE = 1.15 kcal/mol, Table 1) on the 53 unique double and triple mutations and the 104 unique mutations respectively, which demonstrates the robustness of our approach and its applicability to understand mutational effects upon diverse multi-point mutations.

### Performance on additive and synergistic multiple point mutations

From the 38 multiple mutations which have their individual ΔΔGs from single-point mutation dataset, we identified 24 additive and 14 synergistic sets of multiple-point mutations. The mmCSM-AB model was evaluated across both sets, showing comparable performance regardless of whether the mutation effects were additive or synergistic. mmCSM-AB achieved a Pearson's correlation of 0.97 (RMSE = 0.84 kcal/mol) across the multiple point mutations identified as additive, and 0.94 (RMSE = 1.62 kcal/mol) across those identified as synergistic.

### Performance in ranking the effect of multiple point mutations

To guide rational antibody engineering, effective tools need to be able to identify the mutations leading to the greatest improvement in binding affinity. We therefore further assessed the ability of mmCSM-AB and available tools to ranking mutations in the order of most increasing and decreasing binding affinity. mmCSM-AB showed strong performance, achieving the Kendall's Tau and Spearman's rank-correlation coefficient up to 0.71 and 0.86 on the 242 multiple mutations, and outperforming all other approaches (Supplementary Table S3).

### Performance on non-binders

The mmCSM-AB model was further evaluated against the 47 data points where the introduction of multiple mutations led to complete disruption of antigen binding. mmCSM-AB correctly classified 46 out of 47 non-binders (Supplementary Table S4, 98% accuracy).

### Performance on external validation set

We further evaluated the performance of mmCSM-AB using the benchmark dataset from Barlow and colleagues (59). The 59 multiple mutations found in the antibody-antigen complexes did not appear in any other considered dataset and were used for performance comparison of available tools (Table 1). Considering all metrics in Supplementary Table S5, mmCSM-AB showed the second-best performance across 19 comparable methods, achieving a Pearson's correlation of 0.85 (RMSE = 1.66 kcal/mol).

## CONCLUSION

Here we introduce mmCSM-AB, a web server that uses our graph-based signatures to predict the effects of multiple-point missense mutations on antibody binding affinity. The method represents a significant advance upon our current predictive platform, outperforming previous methods, which have primarily been limited to single-point missense proteins. mmCSM-AB can assist antibody design efforts via a freely available, user-friendly and easy to use web server at http://biosig.unimelb.edu.au/mmcsm_ab.

## Supplementary Material

gkaa389_Supplemental_File

## References

[1] Yu J., Kane S., Wu J., Benedettini E., Li D., Reeves C., Innocenti G., Wetzel R., Crosby K., Becker A. et al. Mutation-specific antibodies for the detection of EGFR mutations in non-small-cell lung cancer. Clin. Cancer Res. 2009; 15:3023–3028. 19366827 10.1158/1078-0432.CCR-08-2739

[2] Watt A.D., Crespi G.A., Down R.A., Ascher D.B., Gunn A., Perez K.A., McLean C.A., Villemagne V.L., Parker M.W., Barnham K.J. et al. Do current therapeutic anti-Abeta antibodies for Alzheimer's disease engage the target. Acta Neuropathol. 2014; 127:803–810. 24803227 10.1007/s00401-014-1290-2

[3] Sulea T., Hussack G., Ryan S., Tanha J., Purisima E.O. Application of Assisted Design of Antibody and Protein Therapeutics (ADAPT) improves efficacy of a Clostridium difficile toxin A single-domain antibody. Sci. Rep. 2018; 8:2260. 29396522 10.1038/s41598-018-20599-4 PMC5797146

[4] Almagro J.C., Teplyakov A., Luo J., Sweet R.W., Kodangattil S., Hernandez-Guzman F., Gilliland G.L. Second antibody modeling assessment (AMA-II). Proteins. 2014; 82:1553–1562. 24668560 10.1002/prot.24567

[5] Sircar A., Gray J.J. SnugDock: paratope structural optimization during antibody-antigen docking compensates for errors in antibody homology models. PLoS Comput. Biol. 2010; 6:e1000644. 20098500 10.1371/journal.pcbi.1000644 PMC2800046

[6] Brenke R., Hall D.R., Chuang G.Y., Comeau S.R., Bohnuud T., Beglov D., Schueler-Furman O., Vajda S., Kozakov D. Application of asymmetric statistical potentials to antibody-protein docking. Bioinformatics. 2012; 28:2608–2614. 23053206 10.1093/bioinformatics/bts493 PMC3467743

[7] Cannon D.A., Shan L., Du Q., Shirinian L., Rickert K.W., Rosenthal K.L., Korade M. 3rd, van Vlerken-Ysla L.E., Buchanan A., Vaughan T.J. et al. Experimentally guided computational antibody affinity maturation with de novo docking, modelling and rational design. PLoS Comput. Biol. 2019; 15:e1006980. 31042706 10.1371/journal.pcbi.1006980 PMC6513101

[8] Adolf-Bryfogle J., Kalyuzhniy O., Kubitz M., Weitzner B.D., Hu X., Adachi Y., Schief W.R., Dunbrack R.L. Jr RosettaAntibodyDesign (RAbD): A general framework for computational antibody design. PLoS Comput. Biol. 2018; 14:e1006112. 29702641 10.1371/journal.pcbi.1006112 PMC5942852

[9] Lippow S.M., Wittrup K.D., Tidor B. Computational design of antibody-affinity improvement beyond in vivo maturation. Nat. Biotechnol. 2007; 25:1171–1176. 17891135 10.1038/nbt1336 PMC2803018

[10] Pantazes R.J., Maranas C.D. OptCDR: a general computational method for the design of antibody complementarity determining regions for targeted epitope binding. Protein Eng. Des. Sel. 2010; 23:849–858. 20847101 10.1093/protein/gzq061

[11] Liberis E., Velickovic P., Sormanni P., Vendruscolo M., Lio P. Parapred: antibody paratope prediction using convolutional and recurrent neural networks. Bioinformatics. 2018; 34:2944–2950. 29672675 10.1093/bioinformatics/bty305

[12] Olimpieri P.P., Chailyan A., Tramontano A., Marcatili P. Prediction of site-specific interactions in antibody-antigen complexes: the proABC method and server. Bioinformatics. 2013; 29:2285–2291. 23803466 10.1093/bioinformatics/btt369 PMC3753563

[13] Daberdaku S., Ferrari C. Antibody interface prediction with 3D Zernike descriptors and SVM. Bioinformatics. 2019; 35:1870–1876. 30395191 10.1093/bioinformatics/bty918

[14] Pires D.E., Ascher D.B., Blundell T.L. DUET: a server for predicting effects of mutations on protein stability using an integrated computational approach. Nucleic Acids Res. 2014; 42:W314–W319. 24829462 10.1093/nar/gku411 PMC4086143

[15] Pires D.E., Ascher D.B., Blundell T.L. mCSM: predicting the effects of mutations in proteins using graph-based signatures. Bioinformatics. 2014; 30:335–342. 24281696 10.1093/bioinformatics/btt691 PMC3904523

[16] Pandurangan A.P., Ochoa-Montano B., Ascher D.B., Blundell T.L. SDM: a server for predicting effects of mutations on protein stability. Nucleic Acids Res. 2017; 45:W229–W235. 28525590 10.1093/nar/gkx439 PMC5793720

[17] Rodrigues C.H., Pires D.E., Ascher D.B. DynaMut: predicting the impact of mutations on protein conformation, flexibility and stability. Nucleic Acids Res. 2018; 46:W350–W355. 29718330 10.1093/nar/gky300 PMC6031064

[18] Rodrigues C.H., Ascher D.B., Pires D.E. Kinact: a computational approach for predicting activating missense mutations in protein kinases. Nucleic Acids Res. 2018; 46:W127–W132. 29788456 10.1093/nar/gky375 PMC6031004

[19] Pires D.E., Blundell T.L., Ascher D.B. Platinum: a database of experimentally measured effects of mutations on structurally defined protein-ligand complexes. Nucleic Acids Res. 2015; 43:D387–D391. 25324307 10.1093/nar/gku966 PMC4384026

[20] Pires D.E., Ascher D.B. mCSM-AB: a web server for predicting antibody-antigen affinity changes upon mutation with graph-based signatures. Nucleic Acids Res. 2016; 44:W469–W473. 27216816 10.1093/nar/gkw458 PMC4987957

[21] Pires D.E., Ascher D.B. CSM-lig: a web server for assessing and comparing protein-small molecule affinities. Nucleic Acids Res. 2016; 44:W557–W561. 27151202 10.1093/nar/gkw390 PMC4987933

[22] Pires D.E., Blundell T.L., Ascher D.B. mCSM-lig: quantifying the effects of mutations on protein-small molecule affinity in genetic disease and emergence of drug resistance. Sci. Rep. 2016; 6:29575. 27384129 10.1038/srep29575 PMC4935856

[23] Pires D.E.V., Ascher D.B. mCSM-NA: predicting the effects of mutations on protein-nucleic acids interactions. Nucleic Acids Res. 2017; 45:W241–W246. 28383703 10.1093/nar/gkx236 PMC5570212

[24] Rodrigues C.H.M., Myung Y., Pires D.E.V., Ascher D.B. mCSM-PPI2: predicting the effects of mutations on protein-protein interactions. Nucleic Acids Res. 2019; 47:W338–W344. 31114883 10.1093/nar/gkz383 PMC6602427

[25] Myung Y., Rodrigues C.H.M., Ascher D.B., Pires D.E.V. mCSM-AB2: guiding rational antibody design using graph-based signatures. Bioinformatics. 2020; 36:1453–1459. 31665262 10.1093/bioinformatics/btz779

[26] Jafri M., Wake N.C., Ascher D.B., Pires D.E., Gentle D., Morris M.R., Rattenberry E., Simpson M.A., Trembath R.C., Weber A. et al. Germline mutations in the CDKN2B tumor suppressor gene predispose to renal cell carcinoma. Cancer Discov. 2015; 5:723–729. 25873077 10.1158/2159-8290.CD-14-1096

[27] Usher J.L., Ascher D.B., Pires D.E., Milan A.M., Blundell T.L., Ranganath L.R. Analysis of HGD gene mutations in patients with alkaptonuria from the United Kingdom: Identification of novel mutations. JIMD Rep. 2015; 24:3–11. 25681086 10.1007/8904_2014_380 PMC4582018

[28] Nemethova M., Radvanszky J., Kadasi L., Ascher D.B., Pires D.E., Blundell T.L., Porfirio B., Mannoni A., Santucci A., Milucci L. et al. Twelve novel HGD gene variants identified in 99 alkaptonuria patients: focus on ‘black bone disease’ in Italy. Eur. J. Hum. Genet. 2016; 24:66–72. 25804398 10.1038/ejhg.2015.60 PMC4795215

[29] Soardi F.C., Machado-Silva A., Linhares N.D., Zheng G., Qu Q., Pena H.B., Martins T.M.M., Vieira H.G.S., Pereira N.B., Melo-Minardi R.C. et al. Familial STAG2 germline mutation defines a new human cohesinopathy. NPJ Genom Med. 2017; 2:7. 29263825 10.1038/s41525-017-0009-4 PMC5677968

[30] Trezza A., Bernini A., Langella A., Ascher D.B., Pires D.E.V., Sodi A., Passerini I., Pelo E., Rizzo S., Niccolai N. et al. A computational approach from gene to structure analysis of the human ABCA4 transporter involved in genetic retinal diseases. Invest. Ophthalmol. Vis. Sci. 2017; 58:5320–5328. 29049734 10.1167/iovs.17-22158

[31] Hnizda A., Fabry M., Moriyama T., Pachl P., Kugler M., Brinsa V., Ascher D.B., Carroll W.L., Novak P., Zaliova M. et al. Relapsed acute lymphoblastic leukemia-specific mutations in NT5C2 cluster into hotspots driving intersubunit stimulation. Leukemia. 2018; 32:1393–1403. 29535428 10.1038/s41375-018-0073-5

[32] Ascher D.B., Spiga O., Sekelska M., Pires D.E.V., Bernini A., Tiezzi M., Kralovicova J., Borovska I., Soltysova A., Olsson B. et al. Homogentisate 1,2-dioxygenase (HGD) gene variants, their analysis and genotype-phenotype correlations in the largest cohort of patients with AKU. Eur. J. Hum. Genet. 2019; 27:888–902. 30737480 10.1038/s41431-019-0354-0 PMC6777518

[33] Ascher D.B., Wielens J., Nero T.L., Doughty L., Morton C.J., Parker M.W. Potent hepatitis C inhibitors bind directly to NS5A and reduce its affinity for RNA. Sci. Rep. 2014; 4:4765. 24755925 10.1038/srep04765 PMC3996483

[34] Phelan J., Coll F., McNerney R., Ascher D.B., Pires D.E., Furnham N., Coeck N., Hill-Cawthorne G.A., Nair M.B., Mallard K. et al. Mycobacterium tuberculosis whole genome sequencing and protein structure modelling provides insights into anti-tuberculosis drug resistance. BMC Med. 2016; 14:31. 27005572 10.1186/s12916-016-0575-9 PMC4804620

[35] Hawkey J., Ascher D.B., Judd L.M., Wick R.R., Kostoulias X., Cleland H., Spelman D.W., Padiglione A., Peleg A.Y., Holt K.E. Evolution of carbapenem resistance in Acinetobacter baumannii during a prolonged infection. Microb Genom. 2018; 4:e000165. 10.1099/mgen.0.000165 PMC5885017 29547094

[36] Holt K.E., McAdam P., Thai P.V.K., Thuong N.T.T., Ha D.T.M., Lan N.N., Lan N.H., Nhu N.T.Q., Hai H.T., Ha V.T.N. et al. Frequent transmission of the Mycobacterium tuberculosis Beijing lineage and positive selection for the EsxW Beijing variant in Vietnam. Nat. Genet. 2018; 50:849–856. 29785015 10.1038/s41588-018-0117-9 PMC6143168

[37] Karmakar M., Globan M., Fyfe J.A.M., Stinear T.P., Johnson P.D.R., Holmes N.E., Denholm J.T., Ascher D.B. Analysis of a novel pnca mutation for susceptibility to pyrazinamide therapy. Am. J. Respir. Crit. Care Med. 2018; 198:541–544. 29694240 10.1164/rccm.201712-2572LE PMC6118032

[38] Portelli S., Phelan J.E., Ascher D.B., Clark T.G., Furnham N. Understanding molecular consequences of putative drug resistant mutations in Mycobacterium tuberculosis. Sci. Rep. 2018; 8:15356. 30337649 10.1038/s41598-018-33370-6 PMC6193939

[39] Vedithi S.C., Malhotra S., Das M., Daniel S., Kishore N., George A., Arumugam S., Rajan L., Ebenezer M., Ascher D.B. et al. Structural Implications of Mutations Conferring Rifampin Resistance in Mycobacterium leprae. Sci. Rep. 2018; 8:5016. 29567948 10.1038/s41598-018-23423-1 PMC5864748

[40] Karmakar M., Rodrigues C.H.M., Holt K.E., Dunstan S.J., Denholm J., Ascher D.B. Empirical ways to identify novel Bedaquiline resistance mutations in AtpE. PLoS One. 2019; 14:e0217169. 31141524 10.1371/journal.pone.0217169 PMC6541270

[41] Karmakar M., Rodrigues C.H.M., Horan K., Denholm J.T., Ascher D.B. Structure guided prediction of Pyrazinamide resistance mutations in pncA. Sci. Rep. 2020; 10:1875. 32024884 10.1038/s41598-020-58635-x PMC7002382

[42] Vedithi S.C., Rodrigues C.H.M., Portelli S., Skwark M.J., Das M., Ascher D.B., Blundell T.L., Malhotra S. Computational saturation mutagenesis to predict structural consequences of systematic mutations in the beta subunit of RNA polymerase in Mycobacterium leprae. Comput. Struct. Biotechnol. J. 2020; 18:271–286. 32042379 10.1016/j.csbj.2020.01.002 PMC7000446

[43] Pires D.E., Blundell T.L., Ascher D.B. pkCSM: predicting small-molecule pharmacokinetic and toxicity properties using graph-based signatures. J. Med. Chem. 2015; 58:4066–4072. 25860834 10.1021/acs.jmedchem.5b00104 PMC4434528

[44] Kaminskas L.M., Pires D.E.V., Ascher D.B. dendPoint: a web resource for dendrimer pharmacokinetics investigation and prediction. Sci. Rep. 2019; 9:15465. 31664080 10.1038/s41598-019-51789-3 PMC6820739

[45] Chan L.J., Bulitta J.B., Ascher D.B., Haynes J.M., McLeod V.M., Porter C.J., Williams C.C., Kaminskas L.M. PEGylation does not significantly change the initial intravenous or subcutaneous pharmacokinetics or lymphatic exposure of trastuzumab in rats but increases plasma clearance after subcutaneous administration. Mol. Pharm. 2015; 12:794–809. 25644368 10.1021/mp5006189

[46] Chan L.J., Ascher D.B., Yadav R., Bulitta J.B., Williams C.C., Porter C.J., Landersdorfer C.B., Kaminskas L.M. Conjugation of 10 kDa linear PEG onto trastuzumab Fab' is sufficient to significantly enhance lymphatic exposure while preserving in vitro biological activity. Mol. Pharm. 2016; 13:1229–1241. 26871003 10.1021/acs.molpharmaceut.5b00749

[47] Coelho M.B., Ascher D.B., Gooding C., Lang E., Maude H., Turner D., Llorian M., Pires D.E., Attig J., Smith C.W. Functional interactions between polypyrimidine tract binding protein and PRI peptide ligand containing proteins. Biochem. Soc. Trans. 2016; 44:1058–1065. 27528752 10.1042/BST20160080

[48] Sirin S., Apgar J.R., Bennett E.M., Keating A.E. AB-Bind: Antibody binding mutational database for computational affinity predictions. Protein Sci. 2016; 25:393–409. 26473627 10.1002/pro.2829 PMC4815335

[49] Jemimah S., Yugandhar K., Michael Gromiha M. PROXiMATE: a database of mutant protein-protein complex thermodynamics and kinetics. Bioinformatics. 2017; 33:2787–2788. 28498885 10.1093/bioinformatics/btx312

[50] Jankauskaite J., Jimenez-Garcia B., Dapkunas J., Fernandez-Recio J., Moal I.H. SKEMPI 2.0: an updated benchmark of changes in protein-protein binding energy, kinetics and thermodynamics upon mutation. Bioinformatics. 2019; 35:462–469. 30020414 10.1093/bioinformatics/bty635 PMC6361233

[51] Thiltgen G., Goldstein R.A. Assessing predictors of changes in protein stability upon mutation using self-consistency. PLoS One. 2012; 7:e46084. 23144695 10.1371/journal.pone.0046084 PMC3483175

[52] Barlow K.A., S O.C., Thompson S., Suresh P., Lucas J.E., Heinonen M., Kortemme T. Flex ddG: Rosetta Ensemble-Based estimation of changes in Protein-Protein binding affinity upon mutation. J. Phys. Chem. B. 2018; 122:5389–5399. 29401388 10.1021/acs.jpcb.7b11367 PMC5980710

[53] Webb B., Sali A. Comparative protein structure modeling using MODELLER. Curr Protoc Bioinformatics. 2016; 54:5.6.1–5.6.37. 27322406 10.1002/cpbi.3 PMC5031415

[54] Schymkowitz J., Borg J., Stricher F., Nys R., Rousseau F., Serrano L. The FoldX web server: an online force field. Nucleic Acids Res. 2005; 33:W382–W388. 15980494 10.1093/nar/gki387 PMC1160148

[55] Jubb H.C., Higueruelo A.P., Ochoa-Montano B., Pitt W.R., Ascher D.B., Blundell T.L. Arpeggio: A web server for calculating and visualising interatomic interactions in protein structures. J. Mol. Biol. 2017; 429:365–371. 27964945 10.1016/j.jmb.2016.12.004 PMC5282402

[56] Kabsch W., Sander C. Dictionary of protein secondary structure: pattern recognition of hydrogen-bonded and geometrical features. Biopolymers. 1983; 22:2577–2637. 6667333 10.1002/bip.360221211

[57] Altschul S.F., Madden T.L., Schaffer A.A., Zhang J., Zhang Z., Miller W., Lipman D.J. Gapped BLAST and PSI-BLAST: a new generation of protein database search programs. Nucleic Acids Res. 1997; 25:3389–3402. 9254694 10.1093/nar/25.17.3389 PMC146917

[58] Rose A.S., Bradley A.R., Valasatava Y., Duarte J.M., Prlic A., Rose P.W. NGL viewer: web-based molecular graphics for large complexes. Bioinformatics. 2018; 34:3755–3758. 29850778 10.1093/bioinformatics/bty419 PMC6198858

[59] Jemimah S., Gromiha M.M. Exploring additivity effects of double mutations on the binding affinity of protein-protein complexes. Proteins. 2018; 86:536–547. 29383762 10.1002/prot.25472

