# Supplementary material for: mmCSM-AB: guiding rational antibody engineering through multiple point mutations
Source: Nucleic Acids Res. 2020 May 20;48(W1):W125–31. doi: 10.1093/nar/gkaa389 (PMC7319589; doi:10.1093/nar/gkaa389)
Supplement: gkaa389_Supplemental_File [file gkaa389_supplemental_file.pdf]

SUPPLEMENTARY MATERIAL

mmCSM-AB: guiding rational antibody engineering through multiple point mutations

Yoochan Myung[1,2], Douglas E.V. Pires[1,2,3,*] and David B. Ascher[1,2,4,*]

[1]Computational Biology and Clinical Informatics, Baker Institute, Melbourne, Victoria 3004, Australia
[2]Department of Biochemistry and Molecular Biology, Bio21 Institute, University of Melbourne, Parkville, VIC, 3052, Australia
[3]School of Computing and Information Systems, University of Melbourne, Parkville, VIC, 3052, Australia
[4]Department of Biochemistry, University of Cambridge, Cambridge, CB2 1GA, UK

*To whom correspondence should be addressed D.B.A. Tel: +61 90354794; Email: david.ascher@unimelb.edu.au. Correspondence may also be addressed to D.E.V.P. douglas.pires@unimelb.edu.au.

COMPARATIVE STUDY

For the comparative study, we used standalone scripts to get the binding affinity values for Discovery Studio (1), dFIRE/DFIRE (2), FoldX (3), bASA (4), PRODIGY (5) , and LISA (6), and a webserver, CcharPPI (7), for SIPPER, ZRANK, ZRANK2, FIREDOCK, FIREDOCK_AB, ROSETTADOCK and INSIDE. All structures used in this study were prepared by the FoldX Buildmodel module. The details are described as follows:

- **Discovery Studio:** The changes in binding energy upon multiple mutations were calculated via "Calculate Mutation Energy (Binding)" protocol in Discovery Studio 2018. In this protocol, antigen chain(s) was used as Ligand Chain and the rest of parameters were set to the default.
- **DFIRE / dFIRE:** The changes in DFIRE/dFIRE interaction energy between antibody and antigen chains were calculated as described in Sirin et al (8): All wild-type and mutant complexes were split into complex, antibody and antigen structures to calculate individual energy as the equation (1); then, calculated the difference of interaction energy between wild-type and mutant (2)

$$\Delta G = E(complex) - E(antibody) - E(antigen) \ (1)$$
$$\Delta\Delta G = \Delta G_{wild} - \Delta G_{mutant} \ (2)$$

- **FoldX:** The interaction energy between antibody and antigen groups was calculated by FoldX Analysecomplex module on wild-type and mutant complexes. The change in binding affinity upon mutations was determined by equation (3).

$$\Delta FoldX = FoldX_{wild} - FoldX_{mutant} \ (3)$$

- **bASA:** The buried accessible surface area (bASA) upon mutations was computed using NACCESS as described in Sirin et al (8): firstly, the bASA of wildtype and mutant antibody-antigen complexes were calculated by the equation (4) then subtracted the bASA of mutant from wild (5).

$$bASA = ASA(complex) - ASA(antibody) - ASA(antigen) (4)$$
$$\Delta bASA = bASA_{mutant} - bASA_{wild} \ (5)$$

- **LISA:** The local interaction signal analysis (LISA) uses contact information across the binding interface of antibody-antigen complexes to calculate protein-protein binding affinity. The changes in LISA binding affinity upon mutations were calculated on both wild and mutant antibody-antigen complexes as described by (6).

$$\Delta LISA = LISA_{wild} - LISA_{mutant} \quad (6)$$

- **PRODIGY:** The PRODIGY determines the binding affinity of the protein-protein complex using the number of interfacial contacts and the properties of the non-interacting surface in the complex. The changes in PRODIGY binding affinity were obtained as equation (7).

$$\Delta PRODIGY = PRODIGY_{wild} - PRODIGY_{mutant} \quad (7)$$

- **CcharPPI:** CcharPPI is a webserver that provides over 100 protein-protein interaction-related descriptors as scoring functions which only calculate the energy of a given complex without leading any structural changes. We selected all descriptors in the beginning but filtered top 7 best performing tools, SIPPER, ZRANK, ZRANK2, FIREDOCK, FIREDOCK_AB, ROSETTADOCK, INSIDE to compare with other available tools.

Feature engineering

In the feature engineering step, we calculated different classes of features and evaluated each of the features separately. Then, essential or good performing features were only used to build the final predictive mmCSM-AB model.

- **Energetic terms:** Interaction energy is one of the most commonly used scoring functions for assessing mutational effects. We used the FoldX AnalyseComplex module to calculate interaction energy changes on wild and mutant antibody-antigen complexes.

- **Interatomic interactions:** Atomic interactions such as Hydrogen bond, Ionic, Aromatic, Covalent, VDW, Hydrophobic, Metalsulpur-PI, Amide-Amide, Amide-Ring, PI-PI, Carbon-Pi interactions across antibody-antigen binding interfaces can have significant effects in binding affinity and specificity of antibodies. We implemented Arpeggio (9) to calculate the difference between wild and mutant atomic interactions.

- **Solvent accessible area:** In a protein binding interface, the relative solvent accessible area (RSA) can be a good marker to assess the significance of conformational changes upon mutation. Using DSSP (10), we measured the RSA changes between wild and mutant antibody-antigen complexes.

- **Distance changes:** One of the most distinctive mutational effects in binding interfaces can be measured by distance changes between antibody antigen chains. To avoid getting the distance from the same antibody or antigen chain, we adapted the difference of the closest distance from the mutation site to its binding partner only.

- **Evolutionary score:** In the evolutionary aspect, functionally important sites tend to be conserved to keep the protein stability or protein binding affinity. Using the PSI-BLAST (11), we were able to obtain position specific evolutionary score from Position Specific Scoring Matrix (PSSM) with the following parameters: evolutionary scoring matrix = PAM30, num_iterations = 3, evalue = 1E-10, seg = Yes, comp_based_stats = 1, and db = swissprot.

TABLES

Table S1. Distribution of structure types used in mmCSM-AB.

Type	Count		
	training	blind-test	Sum
Fab	48	26	50
Ab-Ab	1	1	1
Nanobody	3	3	3
Monobody	1	-	1
Others	7	-	7
Total	**60**	**30**	**62**

Table S2. Performance comparison of mmCSM-AB with and without hypothetical reverse mutations.

**training set**	**Performance on 242 multiple mutations**			
	**increasing binding affinity (89 mutations)**		**decreasing binding affinity (153 mutations)**	
	**Pearson (RMSE)**	**Accuracy (number of mis-classified mutations)**	**Pearson (RMSE)**	**Accuracy (number of mis-classified mutations)**
**including hypothetical reverse mutations (1640)**	0.35 (1.19 Kcal/mol)	70% (27)	0.78 (1.36 Kcal/mol)	90% (9)
**only forward mutations (905)**	-0.26 (2.54 Kcal/mol)	25% (67)	0.73 (1.47 Kcal/mol)	92% (7)

Table S3. Comparative performance on correctly ranking mutations in the order of most increasing and decreasing binding affinity.

Method	Performance on 242 multiple mutations					
	**242 (235)[a] overall mutations**		**89 (85)[a] mutations** (increasing affinity)		**153 (144)[a] mutations** (decreasing affinity)	
	Kendall (τ)	Spearman (ρ)	Kendall (τ)	Spearman (ρ)	Kendall (τ)	Spearman (ρ)
**mmCSM_AB**	0.67	0.81	0.50	0.64	0.71	0.86
**mCSM_PPI**	0.51 ***	0.67 ***	0.31 *	0.44	0.41 ***	0.56 ***
**mCSM_PPI2**	0.60 *	0.76	0.30 *	0.43 *	0.46 ***	0.61 ***
**mCSM_AB**	0.56 ***	0.73 *	0.34 *	0.48	0.61 **	0.80
**mCSM_AB2**	0.50 ***	0.64 ***	0.45	0.60	0.54 ***	0.70 ***
**DiscoveryStudio**	0.51 ***	0.67 ***	0.45	0.60	0.54 ***	0.70 ***
**DFIRE**	0.47 ***	0.62 ***	0.47	0.62	0.50 ***	0.65 ***
**bASA**	0.47 ***	0.62 ***	0.37	0.51	0.47 ***	0.63 ***
**dFoldX**	0.51 ***	0.66 ***	0.37	0.50	0.46 ***	0.63 ***
**SIPPER**	0.37 ***	0.51 ***	0.43	0.56	0.48 ***	0.65 ***
**ZRANK**	0.44 ***	0.59 ***	0.49	0.64	0.48 ***	0.63 ***
**dFIRE**	0.47 ***	0.62 ***	0.46	0.63	0.53 ***	0.69 ***
**PRODIGY**	0.44 ***	0.59 ***	0.30 *	0.43 *	0.46 ***	0.61 ***
**FIREDOCK**	0.40 ***	0.54 ***	0.38	0.51	0.54 ***	0.70 ***
**FIREDOCK_AB**	0.40 ***	0.55 ***	0.30 *	0.43 *	0.41 ***	0.57 ***
**ROSETTADOCK**	0.45 ***	0.61 ***	0.43	0.58	0.47 ***	0.63 ***
**ZRANK2**	0.48 ***	0.64 ***	0.36	0.50	0.66	0.83

**INSIDE**	0.37<br>***	0.51<br>***	0.31<br>*	0.44	0.51<br>***	0.68<br>***
**LISA**	0.43<br>***	0.59<br>***	0.36	0.49	0.53<br>***	0.70<br>***

*: p-value <0.05, **: p-value <0.01, ***: p-value <0.001; Statistical significance of Kendall's Tau and Spearman's rank-correlation coefficient was evaluated by Fisher's r-to-z transformation (two-tailed) after Pearson's conversion described from Walker et al. [2003] and Zimmerman et al. [2003]
a: Multiple mutations originated from PDBs which have only one multiple mutation were excluded.

Table S4. Performance of mmCSM-AB on identifying non-binders.

Method	47 non-binders	
	Accuracy	miss classification
**mmCSM_AB**	98%	1
**mCSM_PPI**	100%	0
**mCSM_PPI2**	85%	7
**mCSM_AB**	100%	0
**mCSM_AB2**	64%	17
**DiscoveryStudio**	81%	9
**DFIRE**	19%	38
**bASA**	83%	8
**FoldX**	89%	5
**SIPPER**	26%	35
**ZRANK**	79%	10
**dFIRE**	23%	36
**PRODIGY**	87%	6
**FIREDOCK**	62%	18
**FIREDOCK_AB**	66%	16
**ROSETTADOCK**	85%	7
**ZRANK2**	81%	9
**INSIDE**	55%	21
**LISA**	70%	14

Table S5. Performance comparison of available methods on validation set.

Method	Validation set Barlow et al.		
	Pearson (ρ)	Spearman (ρ)	RMSE
**mmCSM_AB**	0.85	0.88	1.66
**mCSM_PPI**	0.70 *	0.72 *	2.16
**mCSM_PPI2**	0.82	0.84	1.49
**mCSM_AB**	0.80	0.81	1.70
**mCSM_AB2**	0.91	0.92	4.62
**Flex ddG (Barlow et al.)**	0.62 **	0.67 **	5.19
**DiscoveryStudio**	0.53 ***	0.58 ***	2.18
**DFIRE**	0.50 ***	0.57 ***	2.24
**bASA**	0.43 ***	0.48 ***	73.84
**dFoldX**	0.66 *	0.69 **	1.94
**SIPPER**	0.51 ***	0.48 ***	4.11
**ZRANK**	0.34 ***	0.34 ***	6.88
**dFIRE**	0.50 ***	0.57 ***	2.24
**PRODIGY**	0.68 *	0.61 ***	2.62
**FIREDOCK**	0.42 ***	0.39 ***	5.06
**FIREDOCK_AB**	0.46 ***	0.45 ***	4.57
**ROSETTADOCK**	0.51 ***	0.59 ***	2.49
**ZRANK2**	0.53 ***	0.60 ***	28.02

**INSIDE**	0.21 ***	0.22 ***	2.77
**LISA**	0.06 ***	0.11 ***	2.97

*: p-value < 0.05, **: p-value <0.01, ***: p-value <0.001; Statistical significance of Pearson's correlation coefficient was evaluated by Fisher's r-to-z transformation (two-tailed) and Spearman's rank-correlation coefficient was converted as described by Walker et al[2003]. into Pearson's correlation before we applied the transformation.

FIGURES

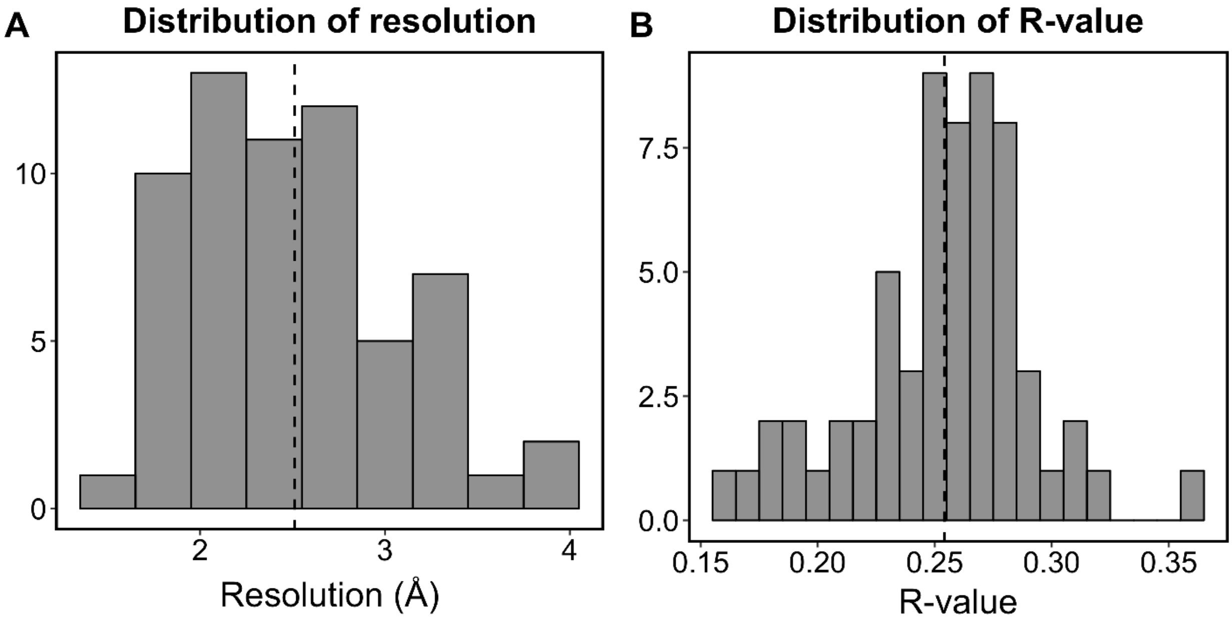

FIGURE S1. Distribution of structural properties. (A) The resolution from 62 3D structures shows an average 2.51 Å (dash line). (B) The quality of atomic model from 61 X-ray structures (1 missing) with an average of 0.25.

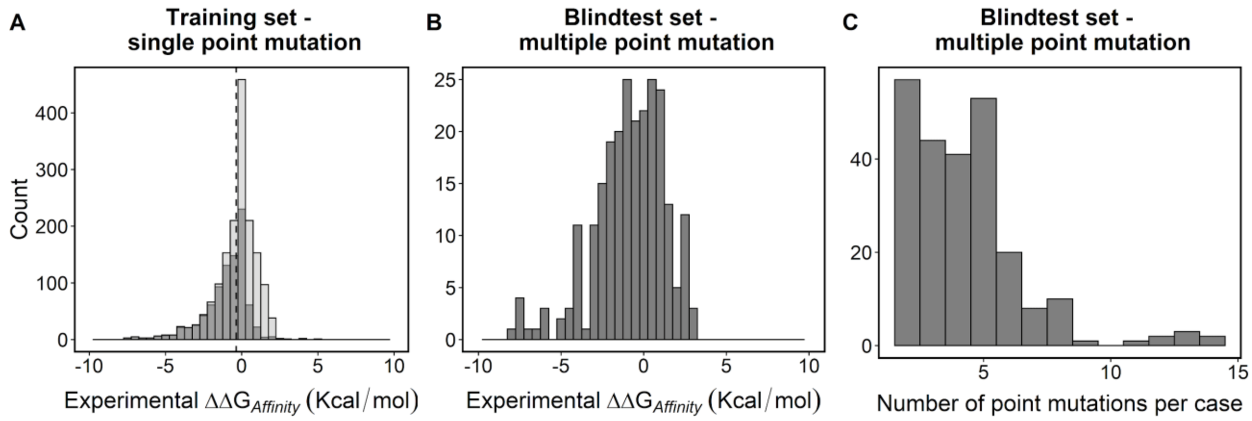

FIGURE S2. Distribution of training and blindtest datasets. (A) The bias of ΔΔG from 905 single-point mutations (dark gray) was balanced out after adding 735 hypothetical reverse mutations (light grey). (B) The 242 multi-point mutation blindtest set shows average and median ΔΔG of -0.86 and -0.62 Kcal/mol, respectively. (C) The double and triple mutations account for about 42 % of all multiple mutations upto 14 mutations per case.

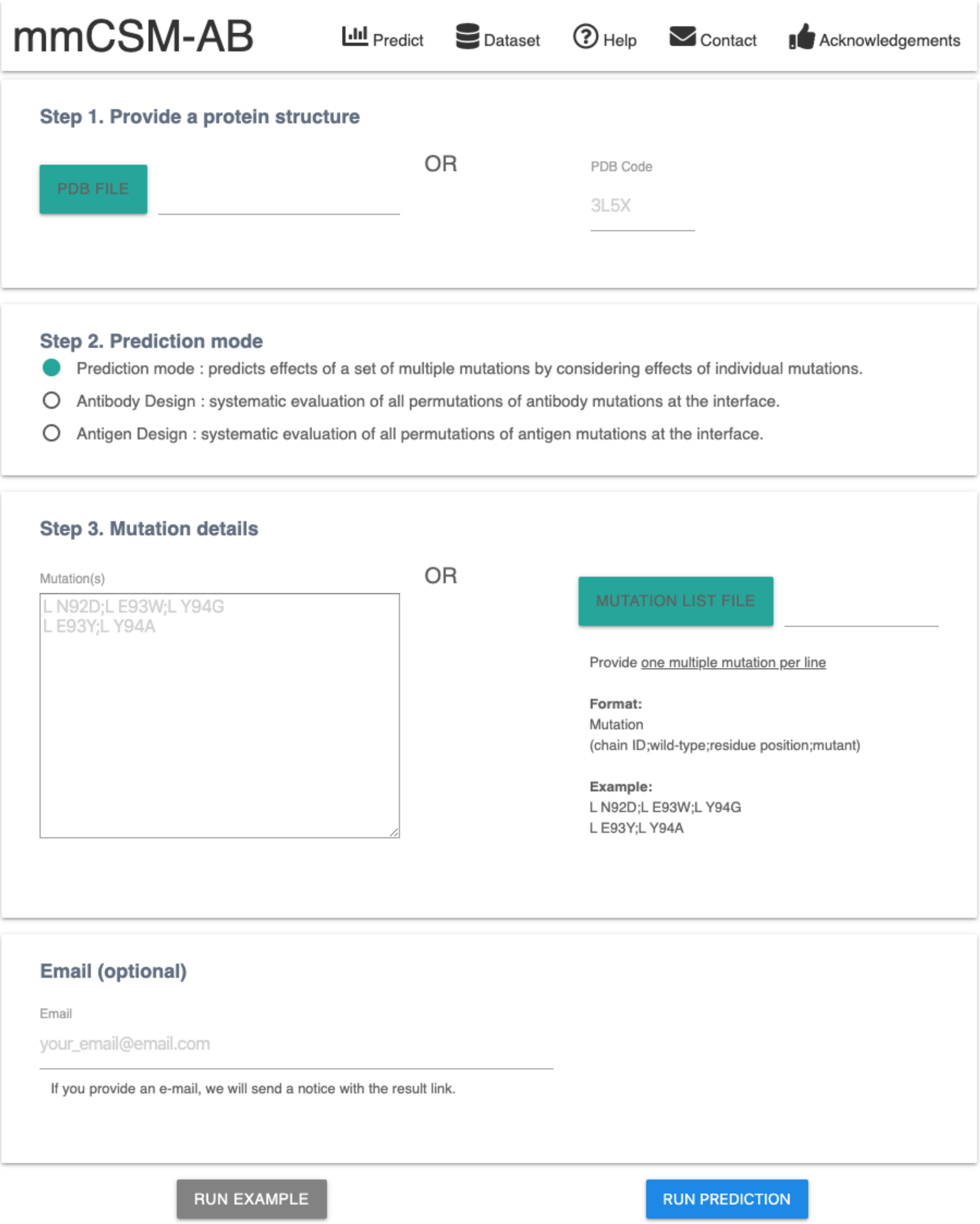

FIGURE S3. Submission page for mmCSM-AB. Users are required to provide an antibody-antigen complex in PDB format, by either uploading a file or providing a PDB accession code. For "Prediction Mode", mutation information can be provided in the text area (with multi-point mutations separated by semicolons) or by submitting a mutation file. For both "Antibody Design" and "Antigen Design" interface residues will be identified and permutations of mutations assessed.

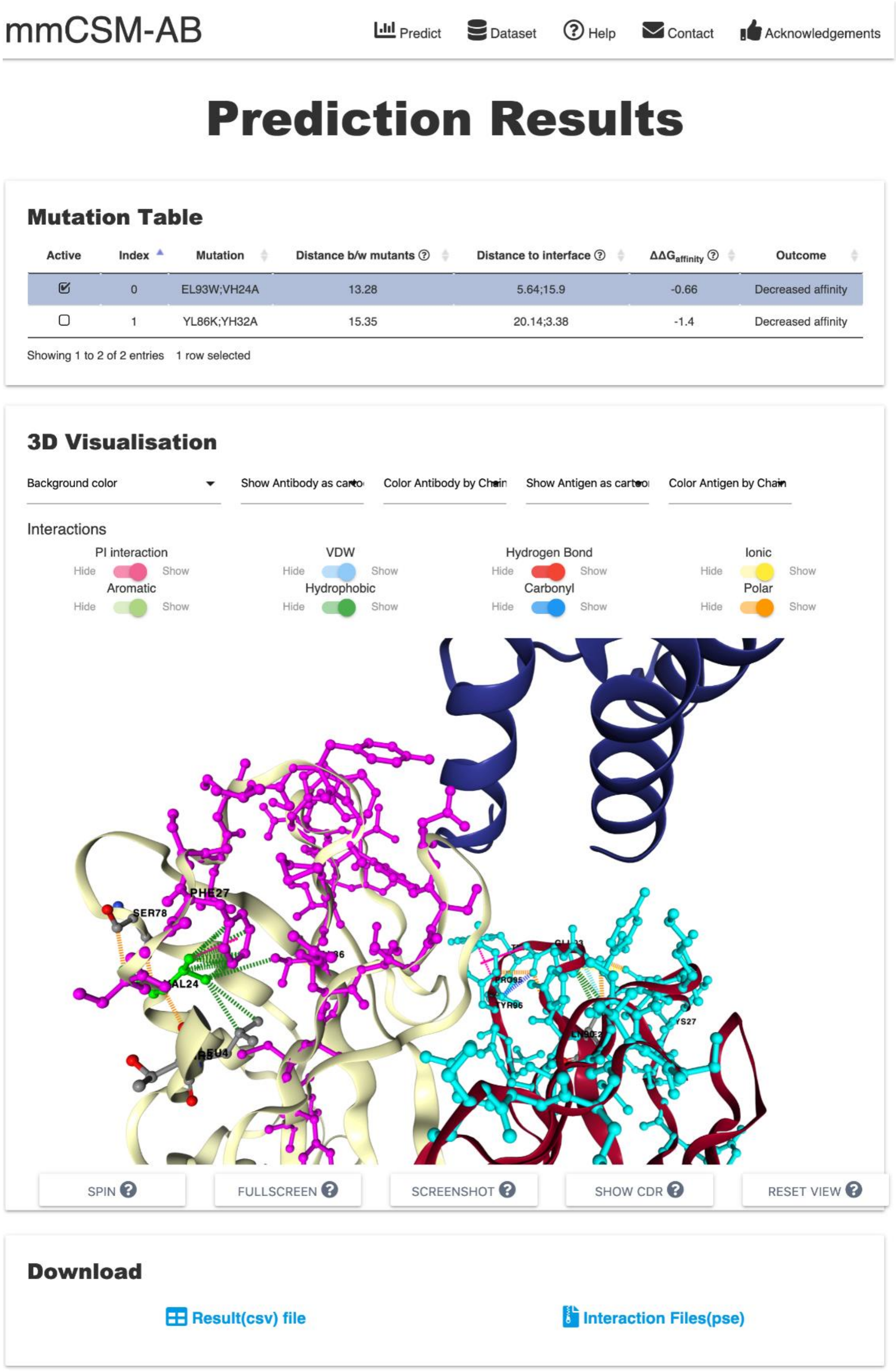

FIGURE S4. Results page for Prediction Mode. The results page provides the predicted effect on Ab-antigen binding (given in Kcal/mol) together with complementary information in a tabular format. Inter-residue atomic interactions involving the wild-type residues can be shown via an interactive 3D viewer. Both prediction data and visualisation files are available for download.

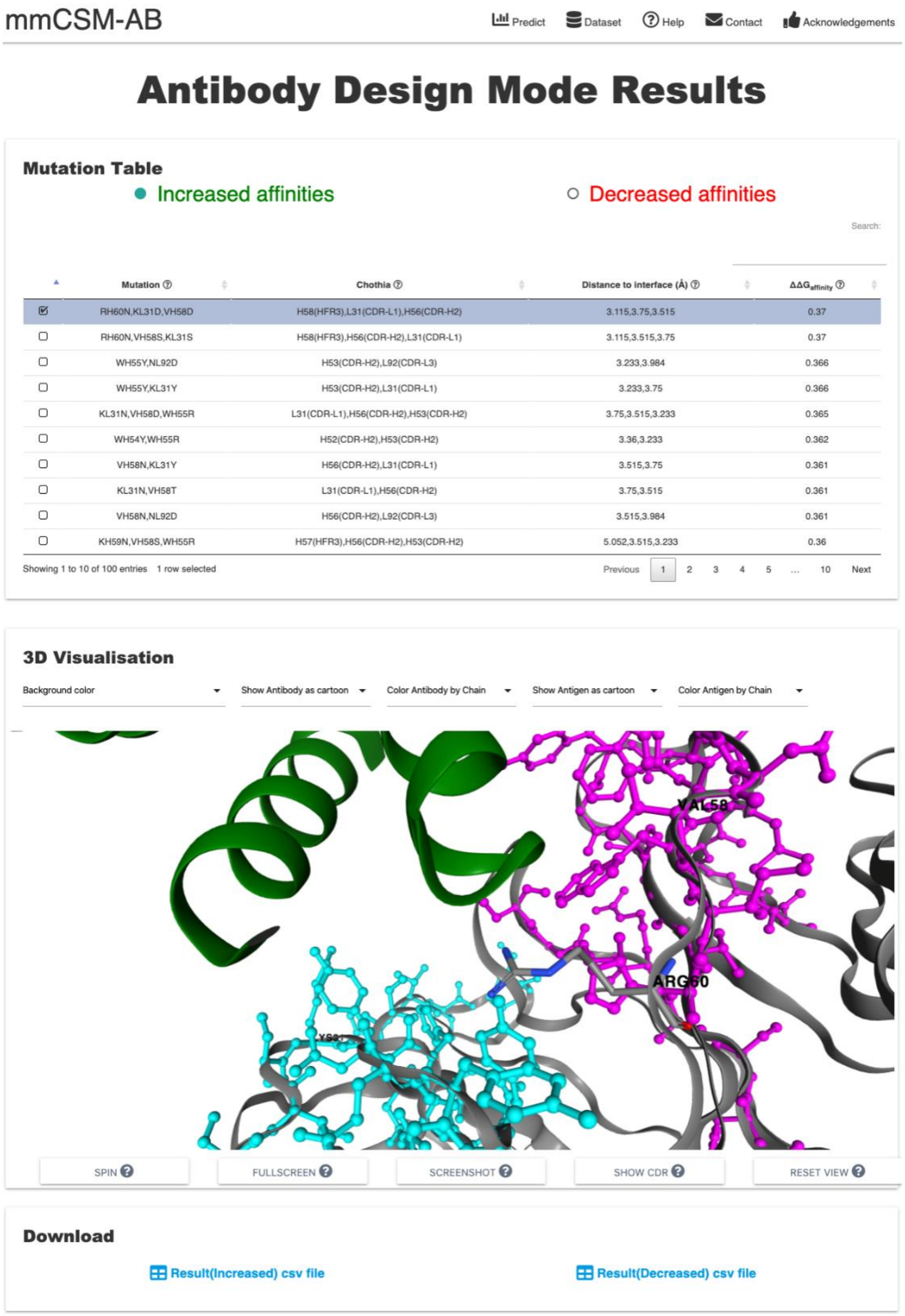

FIGURE S5. Results page for Design Mode. The results page displays the top 100 mutations increasing and decreasing affinities of the ab-antigen, their predicted effect on Ab-antigen binding (given in Kcal/mol) together with complementary information in a tabular format. Inter-residue atomic interactions involving the wild-type residues can be shown via an interactive 3D viewer by selecting each row. Both prediction data and visualisation files are available for download.

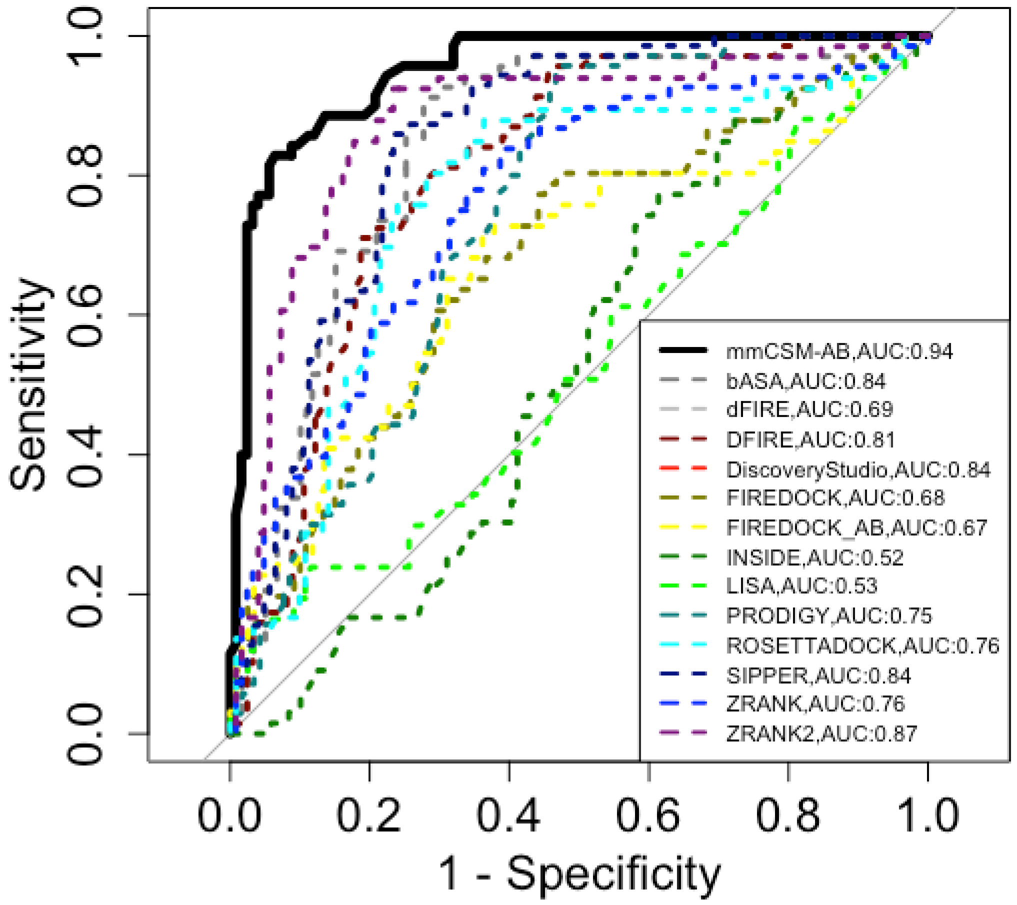

FIGURE S6. Performance of mmCSM-AB on classifying mutations. Performance of mmCSM-AB and other similar methods was assessed by the Area Under the receiver operating characteristic Curves (AUC) in terms of their ability to correctly identify mutations increasing our decreasing affinity. mmCSM-AB achieved an AUC of 0.94, higher than alternative methods.

REFERENCES

1. Inc., A. (2013) Discovery Studio Modeling Environment, release 4.0.
2. Yang, Y. and Zhou, Y. (2008) Specific interactions for ab initio folding of protein terminal regions with secondary structures. *Proteins*, **72**, 793-803. http://dx.doi.org/10.1002/prot.21968
3. Schymkowitz, J., Borg, J., Stricher, F., Nys, R., Rousseau, F. and Serrano, L. (2005) The FoldX web server: an online force field. *Nucleic Acids Res*, **33**, W382-388. http://dx.doi.org/10.1093/nar/gki387
4. Hubbart, S. and Thornton, J.J.T.i.n.c.r.f.t.r. NACCESS (computer program); Department of Biochemistry and Molecular Biology, University College London: London, 1993.
5. Vangone, A. and Bonvin, A.M.J.e. (2015) Contacts-based prediction of binding affinity in protein–protein complexes. **4**, e07454.
6. Raucci, R., Laine, E. and Carbone, A. (2018) Local Interaction Signal Analysis Predicts Protein-Protein Binding Affinity. *Structure*, **26**, 905-915.e904. http://dx.doi.org/https://doi.org/10.1016/j.str.2018.04.006
7. Moal, I.H., Jiménez-García, B. and Fernández-Recio, J.J.B. (2015) CCharPPI web server: computational characterization of protein–protein interactions from structure. **31**, 123-125.
8. Sirin, S., Apgar, J.R., Bennett, E.M. and Keating, A.E. (2016) AB-Bind: Antibody binding mutational database for computational affinity predictions. *Protein Sci*, **25**, 393-409. http://dx.doi.org/10.1002/pro.2829
9. Jubb, H.C., Higueruelo, A.P., Ochoa-Montano, B., Pitt, W.R., Ascher, D.B. and Blundell, T.L. (2017) Arpeggio: A Web Server for Calculating and Visualising Interatomic Interactions in Protein Structures. *J Mol Biol*, **429**, 365-371. http://dx.doi.org/10.1016/j.jmb.2016.12.004
10. Kabsch, W. and Sander, C. (1983) Dictionary of protein secondary structure: pattern recognition of hydrogen-bonded and geometrical features. *Biopolymers*, **22**, 2577-2637. http://dx.doi.org/10.1002/bip.360221211
11. Altschul, S.F., Madden, T.L., Schaffer, A.A., Zhang, J., Zhang, Z., Miller, W. and Lipman, D.J. (1997) Gapped BLAST and PSI-BLAST: a new generation of protein database search programs. *Nucleic Acids Res*, **25**, 3389-3402. http://dx.doi.org/10.1093/nar/25.17.3389
